# Photoperiod influences visceral adiposity and the adipose molecular clock independent of temperature in wild‐derived *Peromyscus leucopus*

**DOI:** 10.1096/fba.2024-00115

**Published:** 2025-04-17

**Authors:** Margaret E. Newport, Paul Wilson, Shanna Lowes, Marthe Behrends, Alexis Coons, Jeff Bowman, Holly E. Bates

**Affiliations:** ^1^ Department of Biology Trent University Peterborough Ontario Canada; ^2^ Wildlife Research and Monitoring Section Ontario Ministry of Natural Resources Peterborough Ontario Canada

**Keywords:** adiposity, circadian rhythm, clock genes, Peromyscus, photoperiod

## Abstract

Physiology is closely synchronized to daily and seasonal light/dark cycles. Humans artificially extend daylight and experience irregular light schedules, resulting in dysregulation of metabolism and body mass. In rodents, winter‐like conditions (cold and short photoperiod) can alter energy balance and adipose tissue mass. To determine if photoperiod alone, independent of temperature, is a strong enough signal to regulate adiposity, we compared the effects of long and short photoperiod at thermoneutrality on adiposity and WAT gene expression in photoperiod‐sensitive, F1 generation wild‐derived adult male white‐footed mice (*Peromyscus leucopus*). Mice were housed in long‐day (16:8 light:dark) or short‐day (8:16 light:dark) photoperiod conditions at thermoneutrality (27°C) for 4 weeks with the extended light being provided through artificial lighting. Photoperiod did not impact body weight or calorie consumption. However, mice housed in long photoperiod with extended artificial light selectively developed greater visceral WAT mass without changing subcutaneous WAT or interscapular BAT mass. This was accompanied by a decrease in *Adrβ3* and *Ucp1* mRNA expression in visceral WAT with no change in *Pgc1a*, *Lpl*, or *Hsl*. Expression of *Per1*, *Per2*, and *Nr1d1* mRNA in visceral WAT differed between long and short photoperiods over time when aligned to circadian time but not onset of darkness, indicating alterations in clock gene expression with photoperiod. These findings suggest that extended photoperiod through artificial light can promote visceral fat accumulation alone, independent of temperature, supporting that artificial light may play a role in obesity.

## Graphical abstract

Physiology is closely synchronized to daily and seasonal light/dark cycles. Wild‐derived white‐footed mice were housed in long‐day (LD – 16L:8D) or short‐day (SD – 8L:16D) photoperiods at thermoneutrality (27°C). LD selectively increased visceral WAT mass and decreased *Adrβ3* and *Ucp1* expression while inducing misalignment of clock genes compared to SD. Thus, extended photoperiod through artificial light can promote visceral fat accumulation alone, independent of temperature, supporting that artificial light may play a role in obesity.

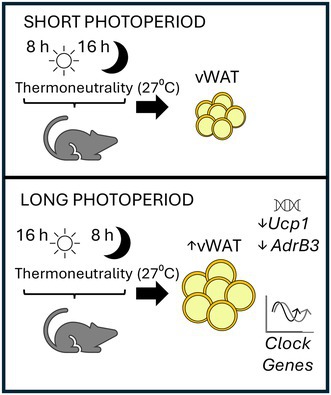

## 1 INTRODUCTION

Circadian dysregulation can contribute to obesity.^1^ Modern humans are exposed to more artificial lighting compared to their ancestors, which has been associated with increased adiposity in rodent^2^ and human^3^, ^4^ studies. Shift workers have increased adiposity^5^ and studies in preclinical/animal models also provide evidence that longer photoperiod (length of daylight) can increase body weight and fat mass.^6^, ^7^, ^8^, ^9^ Thus, environmental photoperiod may contribute to circadian dysregulation and obesity.

Many diurnal behaviors are synchronized to photoperiod (natural daylight), including feeding, sleep, and activity. Photoperiodic information is communicated by the action of clock genes in the suprachiasmatic nucleus (SCN),^10^, ^11^ although circadian‐independent pathways may also play a role.^12^ Light is detected in retinal ganglion cells in the eyes and translated to the SCN, where rhythmic expression of clock genes occurs in an autoregulatory feedback loop.^13^ Core clock genes *Clock* and *Bmal1* are transcription factors for *Per1*, *Per2*, *Per3*, *Cry1*, and *Cry2*, which in turn negatively regulate *Clock* and *Bmal1*.^13^ *Rev‐erbA* (*Nr1d1*) and *Rorα* feedback into the system and aid in maintaining a 24‐h gene expression cycle.^13^ Molecular clocks exist in all cells of the body, including in adipose tissue^14^ and affect physiology because core clock genes act as transcription factors, regulating other genes in a tissue‐specific manner.^15^, ^16^, ^17^ Understanding how clock genes influence physiology is important to fully understand obesity.

Adipose tissue comes in three forms: white adipose tissue (WAT), brown adipose tissue (BAT), and a hybrid type called beige and/or brite adipose tissue, herein referred to as beige. WAT stores excess energy as lipids and is the type of excess fat most associated with obesity.^18^ There are WAT depots throughout the body, both within body cavities as visceral WAT (vWAT: e.g., gonadal fat) and beneath the skin as subcutaneous WAT (scWAT: e.g., inguinal fat).^18^ Individuals with WAT in visceral compartments are at greater risk of developing metabolic syndrome than those who accumulate fat in the subcutaneous regions.^19^ BAT aids in thermoregulation.^20^ Beige adipose tissue is a hybrid with characteristics of both WAT and BAT^21^ and has the capacity for induced thermogenesis.^22^ Beige fat can be induced in WAT depots in inbred lab mice in response to cold exposure or β3‐adrenergic receptor (β3‐AR) agonists but is most prominent in scWAT rather than vWAT.^22^, ^23^, ^24^ The process of thermogenesis in beige and BAT has been a target of obesity research as thermogenesis has the potential to reduce adiposity.^25^

Wild rodents have natural annual variation in their WAT depot sizes, which correlates with seasonal energy demands and food availability.^26^, ^27^ Adipose depots enlarge in summer when nutrients are plentiful and physiology favors energy storage, while in winter, when nutrients are scarce, BAT catabolizes lipids to maintain body temperature and therefore, lipid stores shrink.^26^, ^27^, ^28^ This seasonal physiology synchronizes with environmental temperature and photoperiod.^26^, ^27^, ^28^ These seasonal variations in response to photoperiod and temperature have been studied experimentally in the lab in rodents with winter‐like conditions (low temperature (8°C–20°C) and short photoperiod (8L:16D)) generally causing a reduction in scWAT and vWAT mass, an increase in BAT activity, and an increase in WAT thermogenic genes.^26^, ^27^, ^28^

More recently, studies have tried to determine the strength of photoperiod as a cue to induce thermogenesis in short‐day conditions or increase fat storage in long‐day conditions.^26^, ^28^, ^29^, ^30^, ^31^, ^32^ A reduction in fat depot mass is observed in response to a short photoperiod when studies are conducted at ambient temperatures (generally 20°C–23°C) in deer mice, hamsters, Fischer 344 rats, and voles,^26^, ^28^, ^29^, ^31^, ^32^ temperatures that are notably not at thermoneutrality. Hamsters housed in a short photoperiod at ambient temperature have heightened thermogenic processes, expression of thermogenic genes *Ucp1*, *Pgc1‐α*, and *Adrβ3*, and smaller vWAT depots, despite no change in caloric intake, suggesting that increased thermogenesis may cause the smaller vWAT depot.^26^ Exposure of lab mice to dim light at night at ambient/non‐thermoneutral temperature increases body mass and visceral WAT mass and creates a misalignment of feeding with the time of day by increasing the food ingested during the subjective light phase.^2^ Importantly, all of these studies have been performed at cold or ambient temperatures. Only at thermoneutrality is thermogenesis unnecessary,^33^ suggesting that temperature may have triggered some or all of the responses observed^33^ and housing of animals at thermoneutrality is required to fully isolate the photoperiod cue from environmental temperature. Thus, it is unclear what contribution photoperiod alone plays in the regulation of adipose tissue.

Photoperiod research has evolved to study new laboratory models, since classical lab mice (e.g., C57BL/6) have inbred abnormalities such as melatonin deficiency and/or lack of reproductive regression in response to increased darkness.^34^, ^35^, ^36^ F344 rats demonstrate more reproductive regression, are isogenic, easy to acquire and handle, and have well‐studied behaviors and physiology.^37^, ^38^, ^39^ Outbred models such as hamsters, voles, and deer mice (genus *Peromyscus*) have more natural variability but are more representative of wild populations.^40^ Wild rodent models, such as *Peromyscus*, can be used and produce the most natural response to photoperiod, as they depend on photoperiod for survival.^41^ However, wild models are challenging to work with as age and early life experience may add variability and bias.^42^

In the current study, we aimed to determine the direct impact of photoperiod (independent of temperature) on body weight and feeding behavior, fat accumulation, and visceral WAT gene expression in wild‐derived white‐footed mice (*Peromyscus leucopus*). Specifically, we hypothesized that long photoperiod would cause greater accretion of visceral WAT compared to short photoperiod and that it would do so independent of caloric intake. We bred wild‐caught *P. leucopus* in the lab such that ambient photoperiod, temperature, food availability, predation, maternal care, and age could be controlled^43^ and studied F1 generation offspring to minimize inbreeding and loss of photosensitivity. We examined the impact of photoperiod per se by housing mice at thermoneutrality to remove the temperature cue. We now show that photoperiod can act independently of temperature to selectively regulate visceral WAT accumulation and expression of thermogenic genes (*Ucp1*, *AdrB2*), potentially through shifts in the expression of molecular clock genes (*Per1*, *Per2*, *Nr1d1*). It does so without changing calorie consumption or total body weight gain. These findings provide support that photoperiod (and by extension artificial light) may increase the risk of obesity.

## 2 MATERIALS AND METHODS

### 2.1 Trapping and breeding wild *Peromyscus*

All animal work was approved by the Ontario Ministry of Natural Resources (#1084541) and the Trent University Animal Care Committee (#25281) and followed guidelines set forth by the Canadian Council of Animal Care. Fifteen female and 14 male adult *Peromyscus* were trapped on the Trent University Campus in the summer of 2018. Live traps (H.B. Sherman Inc.) were set at dusk and checked at dawn. Traps were baited with peanut butter, sunflower seeds, and an apple slice, and cotton bedding was provided for warmth. Four trapping locations were used to minimize the risk of in‐breeding once mice are bred in the lab. Off‐target captures were released at trapping sites (i.e., squirrels, chipmunks, and females that were visibly pregnant). Mice were sexed and transported to the Animal Care Facility. Revolution parasiticide (Zoetis, Kirkland, QC, Canada, #: 02242050) was applied as 1 drop behind the ears upon arrival at the animal facility. Animal weights were monitored for 1 month to ensure a healthy transition to the laboratory environment. Once acclimatized to the laboratory setting, mice were genotyped using the *CytB* mitochondrial control region^44^, ^45^ and NCBI BLAST to determine species (*Peromyscus leucopus* or *Peromyscus maniculatus*) (Table S1).^46^, ^47^ Of 27 mice total, 26 were identified as *P. leucopus* and were used to create breeding pairs, and 1 was identified as *P. maniculatus*. To optimize breeding, one male and one female *P. leucopus* from different trapping locations were combined in a cage in long‐day (LD) lighting conditions (16 h light:8 h dark).^48^ 10 breeding pairs in total were established. The body weight of the female mouse was monitored daily to determine if she was pregnant, and breeding pairs were checked daily for pups. When F1 generation pups were born, the breeding pair and pups were moved to a 12:12 lighting schedule to eliminate photoperiod bias. Pups were weaned at 21–24 days old and group housed with same‐sex siblings. Successful breeding pairs remained together and produced serial litters at 12:12 lighting.^48^, ^49^

### 2.2 Animal housing conditions

Mice were housed at 27°C (thermoneutrality)^50^ during all stages of the study (acclimatization, breeding, and before/during the experiment). Thermoneutrality was determined from measurements made during the light phase^50^ and we did not adjust the housing temperature during the dark phase to account for potential diurnal changes in the thermoneutral zone.^51^ Mice were housed in individually ventilated cages (IVC) or conventional housing with external water bottles, provided with a small amount of nesting material (aspen wood shavings and paper) and had a house. Mice had ad libitum access to water and standard mouse chow (Lab Diet Mouse Diet 5015, Richmond, IN, USA; 4.74 kcal/g, 54% carbohydrates, 26% fats, 20% proteins). Lighting conditions used in this study include neutral (12 h light: 12 h dark), long‐day (LD) (16 h light: 8 h dark), and short‐day (SD) (8 h light: 16 h dark). LD and SD photoperiods were synchronized to the onset of the dark phase for ease of measurements. LD and SD groups were housed in the same room, and OttLites® (Tampa, FL, USA) were placed on scheduled timers to artificially extend the photoperiod of the LD group using as much natural lighting as possible.^46^ A dark opaque vinyl curtain separated the LD from the SD group. Lighting levels were confirmed to be similar at various locations on the rack and to not overflow onto the SD group using a light meter.

### 2.3 Experimental protocol (Figure 1)

**FIGURE 1:**
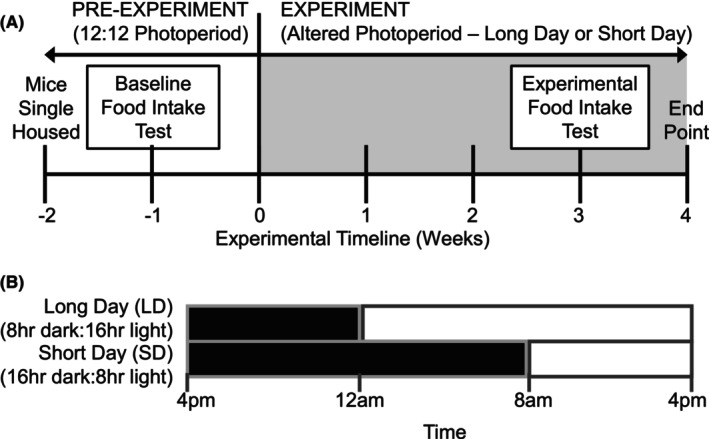
Experimental design (A) Timeline of experiment. Mouse body weight and food consumption were measured weekly. The 24‐h food intake test was done 1 week before the experiment start (baseline) and after 3 weeks in the experiment (experimental). (B) Experimental Photoperiods. Onset of the dark phase at 4 pm was aligned between experimental groups for ease of measurement.

At 10–12 weeks old, lab bred (F1 generation) male mice were singly housed for 2 weeks in 12:12 lighting conditions to acclimatize to single housing and obtain baseline measurements of body weight and food consumption. At 12 weeks old, male mice were divided into LD and SD experimental groups, with siblings distributed equally between groups. Mice were housed in the altered photoperiod (LD or SD) for 4 weeks: the maximum time before short photoperiod induces a reduction in calorie intake in similar rodent species.^26^, ^27^, ^52^ After this point, alterations to metabolism may be indirectly caused by changes in feeding behavior and not direct effects of photoperiod.^26^ Animal weight and food consumption were monitored weekly at the onset of the dark phase to minimize the effects of recent food consumption on body mass. A 24‐h food intake test was conducted at baseline 1 week before beginning the experiment (−1 week) while mice were on 12:12 lighting and after 3 weeks in the altered photoperiod.^27^ For this test, mice were fasted for 5 h prior to the dark phase to ensure they all began the test with a similar baseline state of hunger, and food was returned at the onset of the dark phase. Baseline food consumption was measured after 2, 12 (end of dark phase), and 24 h (end of light phase) by measuring food given and remaining after each time period. Food consumption after 3 weeks of altered photoperiod was measured after 2 h, the end of the dark phase for each group (8 and 16 h), and at the end of the light phase (24 h).

### 2.4 Animal dissection

After 4 weeks of exposure to altered photoperiod, mice were euthanized at 3 time points: CT0 (lights on), CT8 (lights off for SD), and CT16 (lights off for LD) (*n* = 10–12 mice per group). Two mice (one from each group) were euthanized at each time point, alternating which group went first. Mice were euthanized by euthansol injection overdose (50 μL of 340 mg/mL, Merck Animal Health, Kirkland, QC, Canada: #00518816) with decapitation after a surgical plane of anesthesia was confirmed by toe pinch. Trunk blood was collected in a tube containing 50 μL anticoagulant (12 mg/mL EDTA), transferred to a microcentrifuge tube, and centrifuged to separate plasma (1000 ***g*** for 10 min). The visceral epididymal (vWAT) and subcutaneous inguinal (scWAT) white adipose depots, the interscapular brown adipose depot (iBAT), the liver, and testes were collected and weighed. All tissues were flash‐frozen in liquid nitrogen except for left adipose depots, which were preserved for TEM.

### 2.5 Transmission electron microscopy

vWAT of mice (*n* = 4/photoperiod) were analysed using transmission electron microscopy (TEM). Multiple 1‐mm cubes of each sample were fixed (3.0% glutaraldehyde and 1.0% osmium tetroxide), dehydrated (ethanol and propylene oxide dehydration series), infiltrated (graded propylene oxide and EM bed‐Araldite epoxy series), and embedded in resin (EM bed‐Araldite epoxy). The samples were ultrathin sectioned, collected on copper grids, and stained using 5% uranyl acetate and lead citrate. Imaging of vWAT cells was done using a digital camera (AMT‐XR60B, AMT, Woburn, MA, USA) linked to a transmission electron microscope (JEOL 100CX ll Tem Scan, JEOL, Peabody, MA, USA). Length and width of 10 randomly pre‐determined droplets per mouse were measured using AMT‐V600 software to calculate the cross‐sectional area of each lipid droplet, and all lipid droplets in each group were pooled together for statistical analysis of lipid size distribution (*n* = 40 lipid droplets per group).

### 2.6 Visceral epididymal adipose gene expression

Right epididymal fat depots were cryopulverized using liquid nitrogen and a mortar and pestle. RNA was extracted by RNeasy Lipid Tissue Mini Kit (Qiagen #74804, Toronto, ON, Canada). Extracted RNA concentration was measured using a NanoDrop UV–Vis Spectrophotometer (ThermoFisher Scientific: ND‐8000‐GL). cDNA was generated from 1 μg of mRNA by reverse transcription using SuperScript IV Vilo Master Mix with ezDNase treatment (ThermoFisher Scientific #11756050 and #11766051). All RNA samples were reverse transcribed into a positive (RT+) sample (SuperScript IV enzyme) and a negative (RT‐) sample (no SuperScript IV enzyme). RT+ and RT‐ samples were treated with RNaseH (5000 U/mL, New England BioLabs #M0297S) to remove template mRNA from the samples. Relative gene expression was measured in 10 target genes designed using mRNA sequences of *P. leucopus* from NCBI and using Geneious R9 to design and optimize primer and probe sequences (Table 1). Custom primer designs were ordered with FAM‐MGB dye tags (ThermoFisher Scientific #4331348). Real‐time gene expression was analysed from samples using TaqMan™ Fast Advanced Master Mix (ThermoFisher Scientific #4444556) on an Applied Biosystems StepOne™ and StepOnePlus™ Real‐Time PCR System. RT+ and RT‐ samples were run in a 96‐well plate, in duplicate with a “no‐template” negative control. Data quality was evaluated based on standard deviation in the replicate group and a standard deviation >0.5 CT for replicates was re‐run along with the housekeeping gene. Relative gene expression was achieved using the $2^{-\Delta \Delta C_{T}}$ method relative to SD Circadian Time 0 (onset of light) and all target genes were normalized to *Gtf2b*. *Gtf2b* has previously been identified as a quality housekeeping gene for gene expression analysis in all fat depots.^53^ We observed no effect of photoperiod or time on the expression of *Gtf2b* (Figure S1).

**TABLE 1:** qPCR primer and probe sequences.

Gene	Product code (Assay ID)	Forward primer sequence	Probe sequence	Reverse primer sequence	NCBI reference sequence
Gtf2b	APXGRZP	GGACATTTAAAGAAATATGTGCTGTATCTCG	TTGGCCGCTGTTTTAA	CACACTGGTTTCCAAAGCTTTCAAA	XM_028893759.1
Adrb3	APPRPA3	GCCACAGCTGACTTGGTAGT	CACTTTGGCCCTGACTGG	CCACAGCTCGCATCCAGTT	XM_028861480.1
Bmal1	APKA7KC	TGAAAACGTTGAGAGGTGCC	ACCAACCCATACACAGAAGCA	GCTGCCCTGAGAATTAGGTGT	XM_028875865.1
Clock	APKA64D	GATCTCTCAGCCTGCGTCTG	TGGTCCAGATCCCATCCAGT	TCTGTCCTGAGTGAACGTGG	XM_028856884.1
Hsl	APNKVP6	GTGTTGTCGTCCCTGGCTAA	GCATCAACCACTGTGAGGGT	GGTCAGAGGTTAGCGGCATC	XM_028859971.1
Lpl	APMFZ49	AGGGGTCTTGGAGATGTGGA	CTTCATCGACTCCCTGCTGA	CCTTGCTGGGGTTCTCTTCA	XM_028883837.1
Nr1d1	APNKU97	GAGATGCTGTGCGTTTTGGG	CCCCAAGAGAGAGAAGCAGC	CTGCTCAGTTGGTTGTTGGC	XM_028889603.1
Per1	APH6DZE	CACGGTTCTCAGAGGACCAG	CACTCCTGGGTCCGGAAG	CCACACAAGCCATCACGTCA	XM_028869426.1
Per2	APMFZPA	GACCCCATTTGGCTGCTGAT	TGATGACTTACCAGCTGCCC	CCCGGTCCTCTTTCAAGACC	XM_028880350.1
Pgc1a	APGZJFG	CAAGATCAAGGTCCCCAGGC	TCAAGCCACTACAGACACCG	TACAAGGGAGAACTGCGGTG	XM_028865931.1
Ucp1	APZTE33	GATCTCAGCTGGCTTGATGACT	TTGCCCGATGGAATACT	GTCTGACTTTCACGACCTCTGTAG	XM_028881762.1

### 2.7 Statistics

All analyses were done using GraphPad Prism 8 statistics software (Boston, MA, USA). An unbiased cutoff of 2 standard deviations of the mean was used to indicate outliers and removed from analyses. Considering the natural variability of our model, outliers were rare and only 3 out of 37 mice in the LD group and 1 out of 32 mice in the SD group had their weekly body weights and food intake removed. There were no outliers removed from the tissue mass analyses or 24‐h food consumption test. Animal weight, food consumption, and 24 h food intake test measurements were analysed by repeated measures 2‐way ANOVA with Bonferroni correction, with time and photoperiod as the factors being analysed. Epididymal, inguinal, and interscapular fat tissue; and liver and testes weights were normalized to animal body weight on week 4 and analysed using an unpaired *t*‐test. Mean lipid droplet cross‐sectional area was analysed using an unpaired *t*‐test. Distribution of lipid droplet size was analysed using a chi‐squared test for goodness of fit. Relative metabolic and clock gene expression data were analysed using 2‐way ANOVAs with Fisher's LSD test of data points for 0, 8, and 16 h from circadian time (onset of light) and 0, 8, and 16 h from onset of darkness. Time and Photoperiod were the two factors analysed. Figures 4 and 5 include a repetition of the 0‐h data point to provide a full 24‐h gene expression visual but were not included in the statistical analyses. All findings were considered significant when *p* < 0.05.

## 3 RESULTS

**FIGURE 2:**
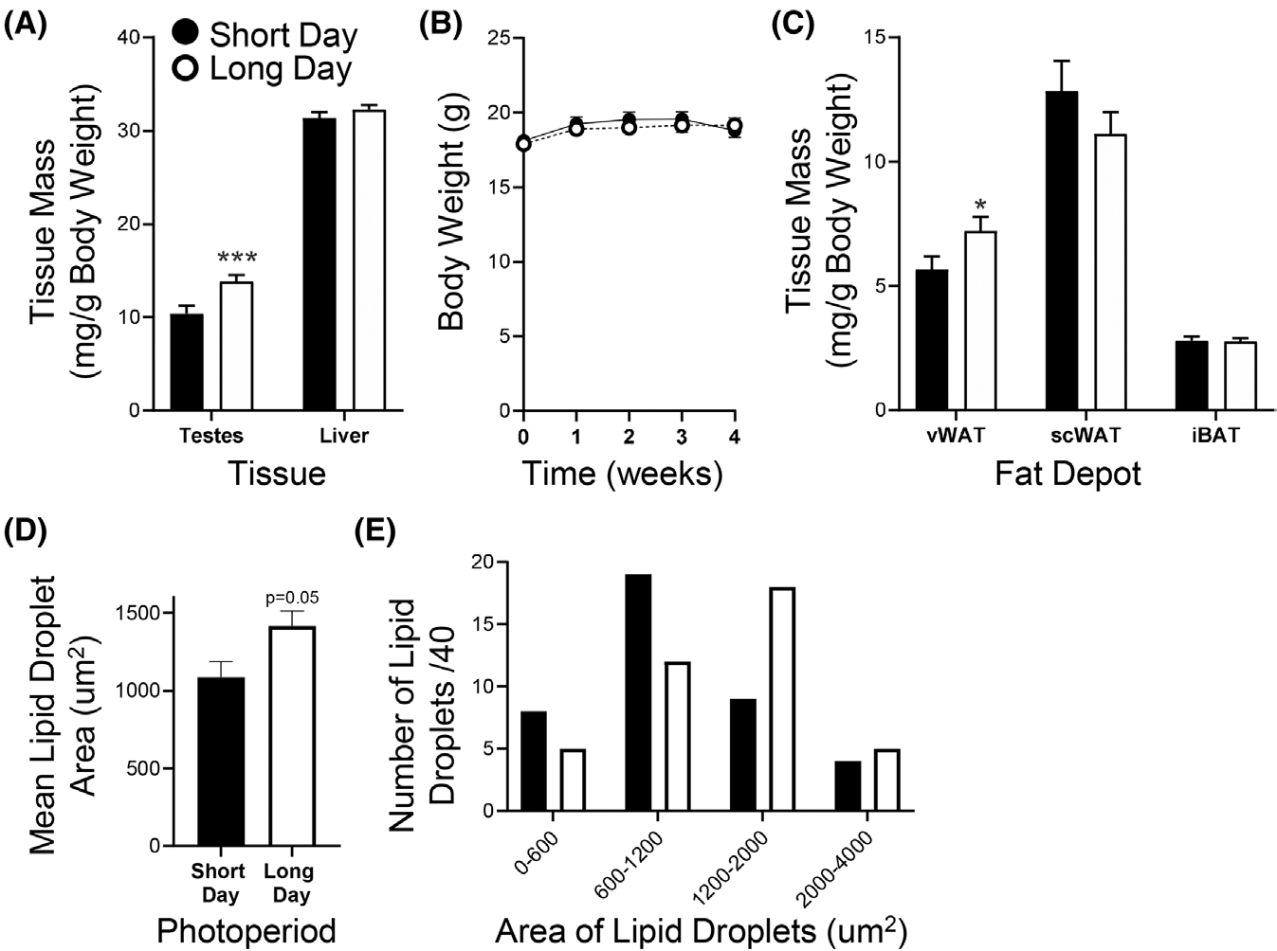
Tissue and body mass. Long photoperiod selectively increases visceral WAT (vWAT) mass. (A) Testes mass is increased after 4 weeks of long photoperiod per se while liver mass is not affected (*n* = 50–52 (testes) and *n* = 28/27 (liver)). (B) Weekly body weight is not affected by photoperiod (*n* = 34/group). (C) vWAT mass is increased after 4 weeks of long day photoperiod while subcutaneous WAT (scWAT) and intrascapular BAT (iBAT) mass are not affected by photoperiod (*n* = 49 Long Day (LD), *n* = 47 Short Day (SD)). (D) Long photoperiod increases the mean size of lipid droplets in vWAT analysed by TEM. (*n* = 4 mice/group with 10 droplets per mouse analysed). (E) Long photoperiod leads to proportionally fewer small lipid droplets (<1200 um^2^) (*p* < 0.05 chi‐squared test). Data is expressed as mean ± SEM. *p < 0.05, ***p < 0.005 versus LD.

Mice demonstrated gonadal regression in response to short photoperiod, as SD testes were 33% lighter than LD testes (*p* = 0.002) (Figure 2A). There were no differences in liver weight between groups, indicating that this effect of photoperiod was organ‐specific (Figure 2A).

Four weeks of altered photoperiod at thermoneutrality did not change body weight (Figure 2B), with mean body weights after 4 weeks of altered photoperiod being nearly identical (LD: 19.1 ± 0.5 g and SD: 18.8 ± 0.5 g). However, long photoperiod affected adipose mass in a depot‐specific manner (Figure 2C) whereby vWAT mass was greater in LD compared to SD housed mice (*p* = 0.049) while the mass of scWAT and iBAT depots was not impacted by photoperiod. Adipocytes in vWAT from LD mice also had an increase in mean lipid droplet area (Figure 2D) (*p* = 0.05) and a change in the size distribution of these lipid droplets, with fewer small droplets and more large droplets compared to SD (Figure 2E) (*p* = 0.01).

**FIGURE 3:**
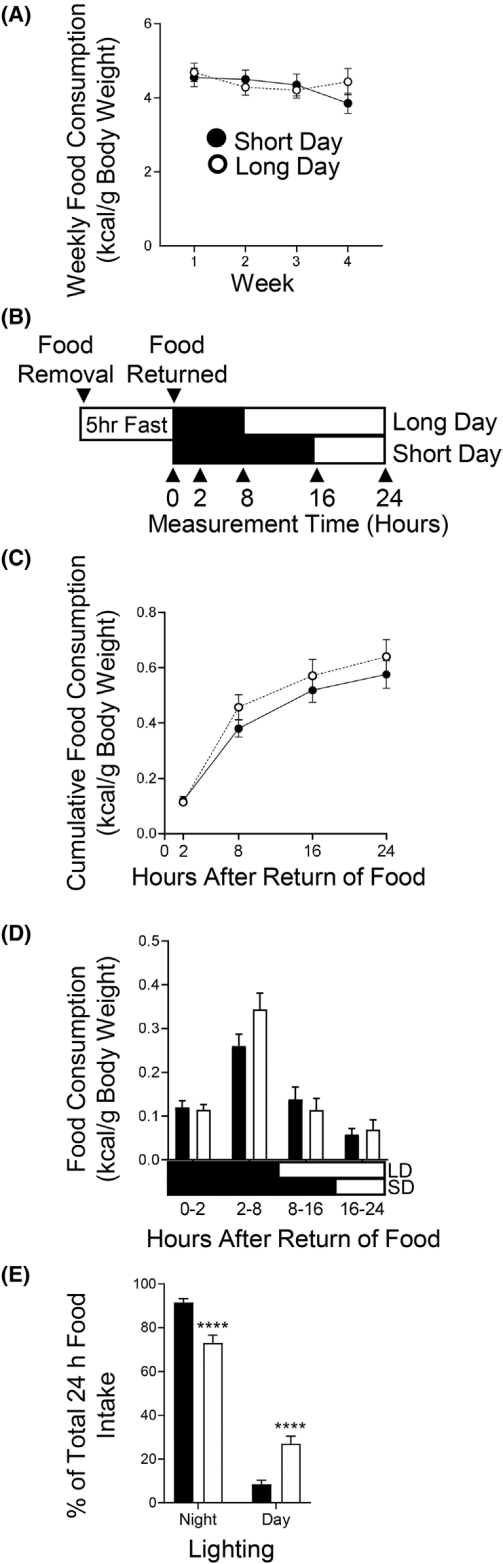
Photoperiod does not affect calorie intake or feeding behavior. (A) Weekly calorie consumption was not affected by altered photoperiod (*n* = 34/group) (B) 24 h Fasting‐Refeeding Food Intake Test Design. Mice were fasted for 5 h before the food intake test began. Food was returned at the onset of dark (time 0). Food consumed was measured after 2, 8, 16, and 24 h. (C) 24‐h cumulative food intake is not altered by photoperiod (*p* = 0.3782) (*n* = 34/group). (D) 24‐h feeding behavior is not significantly altered by photoperiod (*n* = 34/group). (E) Percentage of feeding during the normal feeding/active dark phase versus light phase. Data is expressed as mean ± SEM. ****p < 0.0001 versus Short Day.

Four weeks of altered photoperiod at thermoneutrality did not affect weekly ad libitum food consumption (Figure 3A). Baseline 24‐h feeding behavior was identical between the two groups (Figure S2) and after 3 weeks of altered photoperiod, neither the total calories consumed in 24 h nor in the first 2 h were different (Figure 3C,D). There was a trend for long photoperiod to increase food consumption during the first part of their normal feeding/active (dark) phase (2–8 h after onset of the dark phase, *p* = 0.067) (Figure 3D) and LD mice consumed 3 times more food during their respective light phase (26% in hours 8–24) compared to SD mice (8.5% in hours 16–24) (*p* < 0.0001) (Figure 3E). There was no effect of photoperiod when food consumption was expressed as a percent of the total food consumption or as a rate of food consumption (Figure S3).

**FIGURE 4:**
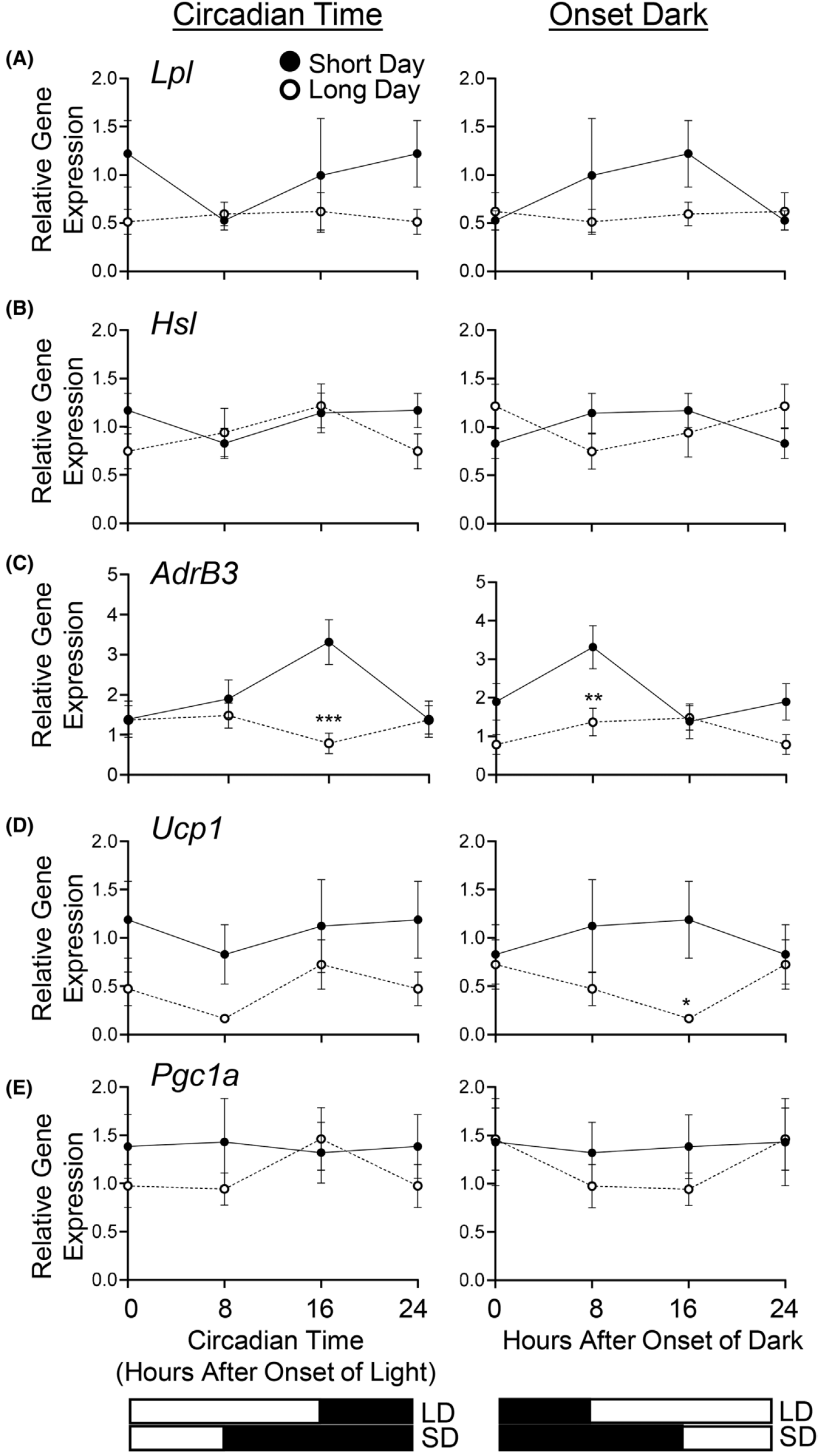
Long photoperiod decreases *Adrβ3* and *Ucp1* mRNA expression in visceral WAT (vWAT). Photoperiod did not alter the expression of *Lpl*, *Hsl*, or *Pgc1a* mRNA expression (*p* > 0.05) but levels of *AdrB3 and Ucp1* were decreased with long photoperiod (main effect photoperiod *p* < 0.05). Graphs were extrapolated to 24 h using data at 0 h to provide a full 24‐h profile. 2‐way ANOVAs were performed without the inclusion of these extrapolated values. Data is expressed as mean ± SEM. *p < 0.05 versus Short Day, **p < 0.001 versus Short Day, ***p < 0.0001 versus Short Day. Long Day (LD), Short Day (SD).

Expression of several fat metabolism genes was measured in vWAT obtained in 8‐h increments and aligned with circadian time (CT0, onset of light) (left panel Figure 4) or onset of dark/feeding (right panel Figure 4). Only *AdrB3* expression varied over time when aligned to the onset of the dark/feeding phase (*p* = 0.03) but not CT0 (Figure 4C). Neither *Lpl*, *Hsl*, nor *Pgc1a* expression was affected by photoperiod, regardless of whether they were aligned to CT0 or onset of dark (Figure 4A,B,E). In contrast, photoperiod affected both *Adrβ3* and *Ucp1* expression (Main Effect Photoperiod *p* = 0.0046 and *p* = 0.02, respectively) with long photoperiod lowering the expression of both genes compared to short photoperiod (Figure 4C,D).

**FIGURE 5:**
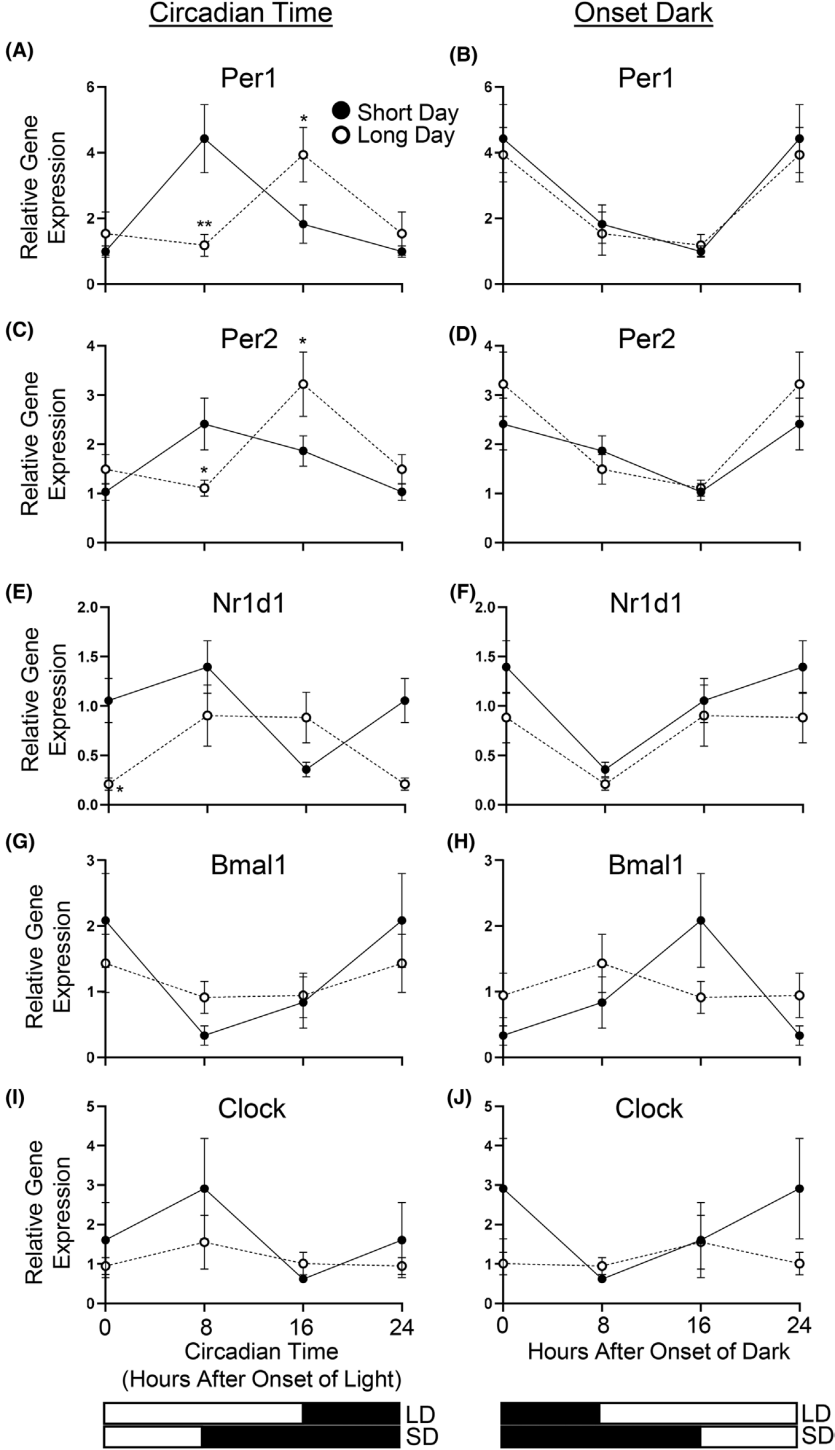
Clock gene expression in visceral WAT (vWAT) when aligned to circadian time (CT0) (A, C, E, G, I) or onset of darkness (B, D, F, H, J). *Per1* and *Per2* mRNA expression is altered by photoperiod over time when aligned to CT0 (A, C) but not onset of darkness (B, D). *Nr1d1* mRNA expression differs with altered photoperiod over time when aligned to CT0 (I) but not onset of darkness (J). *Bmal1* and *Clock* mRNA expression are not altered by photoperiod when aligned to CT0 (G, I) or onset of dark (H, J). Graphs were extrapolated to 24‐h using data at 0‐h to provide a full 24‐h profile. 2‐way ANOVAs were performed without the inclusion of these extrapolated values. Data is mean ± SEM. *p < 0.05, **p < 0.01 versus Short Day. Long Day (LD), Short Day (SD).

Clock genes are thought to play regulatory roles in adipose tissue physiology.^54^ The expression of clock genes *Per1*, *Per2*, *Bmal1*, *Clock*, *and Nr1d1* was measured in vWAT at 8‐h increments (onset of dark/feeding; onset of light for each of the photoperiods, 8 and 16 h after onset of dark) to allow for the circadian variability in their expression. All clock genes measured, except for *Clock*, demonstrated variability over time (Main Effect Time *p* < 0.05). When aligned to CT0, both *Per1* and *Per2* mRNA expression were impacted by photoperiod (Photoperiod × Time *p* = 0.0006 and *p* = 0.004, respectively) (Figure 5A,C), with the highest expression separated by 8 h between photoperiods. However, when aligned to the onset of dark/feeding, the highest expression of both *Per1* and *Per2* occurred at the onset of the dark phase in both groups, with the lowest expression occurring 16 h later such that the effect of photoperiod over time was lost (Figure 5B,D). Gene expression of *Nr1d1* (Reverbα) was affected by photoperiod over time when aligned to CT0 (Time × Photoperiod interaction *p* = 0.01) (Figure 5E). Like *Per1* and *Per2*, the effect of photoperiod over time on *Nr1d1* expression was lost when synchronized with the onset of dark/feeding (Time × Photoperiod *p* = 0.6591) (Figure 5F). When aligned to CT0, *Bmal1* expression showed the highest expression at the onset of light (Figure 5G) but levels were not affected by photoperiod. High variation in *Clock* expression did not lead to changes in expression over time (Figure 5I,J). Mice housed on a 12:12 light:dark cycle have *Clock* and *Bmal1* expression antiphase to *Per1* and *Per2*.^14^ Therefore, we compared the expression of these genes when aligned to the onset of the dark/feeding phase, to which *Per1* and *Per2* appeared to be highly synchronized (Figure 5H,J). *Clock* gene expression showed minimal variability over time, whereas *Bmal1* expression showed a trend to change over time in response to photoperiod (Time × Photoperiod interaction *p* = 0.065). The highest expression of *Bmal1* appeared to synchronize with the onset of the light phase and thus the trough of *Per1* and *Per2*, but was separated by 8 h between photoperiods.

## 4 DISCUSSION

Photoperiod is becoming increasingly recognized as an important regulator of metabolic function,^7^, ^55^ and disruption of circadian rhythms through artificial light may contribute to many metabolic health conditions, including obesity.^4^ Long photoperiod in combination with non‐thermoneutral temperatures can increase body weight and/or fat mass.^26^, ^28^, ^29^, ^30^, ^31^, ^32^, ^56^ In our wild‐derived photosensitive rodent model: F1 generation white‐footed mice (*Peromyscus leucopus*), long photoperiod at thermoneutrality had more selective and subtle effects. Long photoperiod increased the mass of vWAT without affecting scWAT mass, iBAT mass, caloric intake, or body weight. This was accompanied by an increase in vWAT lipid droplet size. Since small lipid droplets are optimized for lipid release while large lipid droplets are optimized for lipid storage,^57^ this shift in droplet size suggests a change in vWAT function with photoperiod. This was accompanied by alterations in vWAT clock gene expression and decreased expression of *AdrB3* and *Ucp1*. Thus, long photoperiod alone, without simultaneous temperature cues, was a strong enough environmental signal to selectively induce visceral adipose accumulation but not an increase in body weight. This novel finding extends many similar studies that observed fat mass changes in response to photoperiod in deer mice, hamsters, voles, mice, and rats, but all at temperatures below thermoneutrality that were unable to fully separate the effects of photoperiod per se from those of temperature.^6^, ^26^, ^28^, ^29^, ^30^, ^31^, ^32^

Despite an increase in visceral adiposity due to long photoperiod, we observed no significant effect of photoperiod on body weight in *P. leucopus* mice housed at thermoneutrality. Body weights in closely related deer mice (*P. maniculatus*) were also not affected by photoperiod at ambient temperature (20°C) although vWAT mass was similarly impacted.^28^ This contrasts with studies in Siberian and Desert hamsters in which greater body weight with long photoperiod at ambient temperatures (20°C) was observed.^26^, ^31^ In Siberian hamsters, long photoperiod at ambient temperature (20°C) also increased fat depot mass non‐selectively, with greater vWAT, scWAT, and iBAT mass compared to short photoperiod.^26^ Cold exposure (4°C–6°C) of inbred lab mice without a change in photoperiod (12:12 light: dark schedule) dramatically lowers scWAT mass and causes more beiging than in the vWAT depot.^22^, ^23^, ^58^, ^59^ Taken together with our findings, this suggests that a temperature cue is necessary to affect scWAT and iBAT mass and that vWAT may be more photoperiod sensitive and scWAT/iBAT more temperature sensitive. The combination of short photoperiod and cold temperature may together cause the low‐fat mass observed in rodents in winter.^26^, ^27^, ^60^

Altered photoperiod did not affect weekly ad libitum caloric intake during the 4‐week study in *P. leucopus*, similar to what has been observed in shorter duration photoperiod studies at 20°C–22°C in seasonal and non‐seasonal rodents.^6^, ^29^, ^31^, ^61^ In contrast, in longer studies in seasonal Siberian Hamsters (10 weeks), a more dramatic reduction in body weight and fat mass in response to shortened photoperiod and non‐thermoneutral temperature takes place^26^, ^52^ which is after the point at which food intake is reduced in response to photoperiod. Inbred lab mice, hamsters, voles, and *Peromyscus* have nocturnal feeding behavior.^48^, ^49^ The 24‐h fasting‐refeeding food intake test revealed that feeding behavior was not affected by 3 weeks of altered photoperiod. Interestingly, this means that during the 8 h when the groups were in different lighting (8–16 h after dark onset, lights on for LD and off for SD), mice were eating a similar amount of food. As feeding and light are both zeitgebers that entrain circadian rhythms, there could be a mismatch during this period between groups that could contribute to circadian dysregulation and differences in fat accumulation.^6^, ^62^, ^63^, ^64^, ^65^ Mice exposed to dim light at night or long photoperiod similarly eat a larger proportion of their food during the daylight hours compared to those without dim light at night, and this occurs with increases in white adipose mass.^2^, ^6^ In our study, *P. leucopus* mice housed in long photoperiod consumed 3 times more calories during their 16‐h light phase than short photoperiod housed mice during their 8‐h light phase, in only twice the time. This may create a disconnect between photoperiod signals and feeding cues to peripheral tissues. Indeed, when mice exposed to altered photoperiods have their food intake restricted to only the first 6 h of the dark phase (time restricted feeding), equalizing the timing of feeding between photoperiods, many metabolic differences are abolished.^6^ Daytime feeding of inbred lab mice can increase adiposity without changing daily caloric intake, and their obesity can be reversed when food is restricted to the dark phase (their natural feeding time).^66^, ^67^ Thus, as suggested,^6^ extended photoperiod may naturally be contributing to this phenomenon with the misalignment of feeding behavior and photoperiod, causing increased visceral adiposity.

Photoperiod has been proposed to be a seasonal light cue that triggers preparation for seasonal changes in temperature, as photoperiod typically changes before the temperature in the natural environment.^68^ We now show that photoperiod can regulate the expression of *Adrβ3* and *Ucp1* in vWAT at thermoneutrality, with long photoperiod decreasing the expression of both transcripts. This suggests that β3‐AR sensitivity may depend at least partially on photoperiod, with short days increasing β3‐AR sensitivity to favor thermogenic/lipolytic pathways and long days lowering β3‐AR sensitivity to favor lipid accumulation. In support of this theory, we observed a shift in the size of lipid droplets from small to large in vWAT of long photoperiod‐housed mice, suggestive of lipid accumulation. Glucose tolerance and thermogenesis are also reduced in light compared to dark through a β3‐AR‐dependent pathway.^69^ Indeed, the importance of sympathetic regulation in mediating the effects of photoperiod on adipose tissue depots is well established, albeit at non‐thermoneutral temperatures. Siberian hamsters housed in long photoperiod experience more dramatic increases in epididymal than in inguinal WAT mass.^70^ Visceral WAT from these hamsters has lower norepinephrine turnover^71^ and decreased β3‐AR mRNA expression^26^ than short photoperiod‐housed hamsters. This is coupled with lower NE‐induced lipolysis^72^ and greater LPL activity.^70^ Furthermore, β3‐AR protein activation by the sympathetic nervous system increases lipid mobilization through post‐translational (phosphorylation) activation of HSL,^73^ levels of which are lower in Siberian hamsters housed in long photoperiod.^24^ We did not observe an effect of photoperiod on *Hsl* or *Lpl* mRNA levels, although this may reflect that HSL is regulated post‐translationally by photoperiod^73^ and that LPL is highly post‐transcriptionally regulated.^74^ Thus, our data is consistent with the theory that long photoperiod slows lipid mobilization and accentuates lipid storage through reduced sympathetic drive and extends this knowledge by illustrating that photoperiod is likely able to do so at least in part without the presence of a temperature cue.

In a thermoneutral environment, as was used in this study, thermogenesis is theoretically not required in beige and brown fat to maintain body temperature.^33^, ^68^ Remarkably, despite this, we observed lower *Ucp1* mRNA levels in vWAT of mice housed in long photoperiods, suggesting that at least at the level of gene expression, UCP1 may be regulated by photoperiod alone and does not require a parallel temperature cue. This is consistent with some^24^, ^75^ but not all^29^ studies that have examined the impact of photoperiod on UCP1 expression in WAT at non‐thermoneutral conditions. Without a direct comparison to mice housed in short photoperiods and lower temperatures, we do not know what the relative contribution is of photoperiod to the full induction of *Ucp1* gene expression, although it is likely that it is much less than that induced by colder temperatures. Adaptive thermogenesis in brown and beige fat is often accompanied by an increase in *Pgc1α* mRNA.^23^ The lack of effect of altered photoperiod on *Pgc1α* mRNA levels in our study suggests that mitochondrial biogenesis, which is regulated by Pgc1a,^76^ may be unaffected when a temperature cue is lacking. Future studies in which other markers of beige cells are measured will be necessary to determine the full contribution of photoperiod per se to beiging of vWAT. In hamsters, 5 weeks of short photoperiod at temperatures below thermoneutrality (20°C) did increase *Pgc1α* mRNA expression in conjunction with *Ucp1* and *Adrβ3* mRNA in epididymal WAT.^26^ Thus, these data and our own suggest that short photoperiod alone, without a simultaneous temperature‐mediated cue, may not be a strong enough trigger for an increase in *Pgc1α* mRNA expression in vWAT and that vWAT cells may be primed for β3‐AR sensitivity should the temperature cue arrive and activate *Ppar* mediated pathways.

Our data shows that short photoperiod per se decreases both *AdrB3* and *Ucp1* gene expression, which raises the possibility that thermogenesis may also be impacted by photoperiod, although this was not measured in the current study. However, other adrenoreceptor subtypes (e.g., β1‐AR) aside from *AdrB3* may also play a role.^77^, ^78^, ^79^ Sympathetic outflow is an important mediator of differential vWAT loss over scWAT loss early in calorie restriction weight loss.^80^ Therefore, it is possible that previous short photoperiod studies in rodents housed below thermoneutrality may result in the activation of multiple β‐AR pathways from the combined photoperiod and temperature cues to cause more dramatic lipolysis, thermogenesis, and vWAT mass changes than photoperiod alone.^26^, ^28^, ^29^, ^30^, ^31^, ^32^ Future studies should investigate the impact of photoperiod on sympathetic drive as well as the balance of the other adrenoreceptor subtypes (β1, β2, α‐2) in vWAT, as these receptors likely also play important roles in lipolysis and beiging from adipose progenitor cells.

Clock genes participate in an autoregulatory feedback loop that synchronizes their expression to a 24‐h cycle, and in 12L:12D studies of lab mouse adipose tissues, both *Per1*/*Per2* and *Nr1d1* mRNA expression are antiphase to *Clock* and *Bmal1*.^14^ We measured clock gene expression every 8 h at only three times to capture expression at lights off and lights on, and did not have a 12:12 light: dark photoperiod control group. Therefore, we did not have enough data points nor the appropriate control group for detailed analyses of circadian rhythmicity. In our study in *P. leucopus*, the *Per1* and *Per2* genes in vWAT had the highest expression at the onset of dark, and *Bmal1* had the highest expression with the onset of light, as observed in lab mice.^14^ *Nr1d1* also had the highest expression at the onset of dark. When synchronized to what appeared to be the most appropriate zeitgeber (onset of dark for *Per1*, *Per2*, *Nr1d1* and onset of light for *Bmal1* and *Clock*), none of the clock genes measured experienced differences in the absolute level of their expression in response to altered photoperiod. Importantly, since the onset of light was 8‐(LD) or 16‐(SD) h following the onset of dark, this suggests that altered photoperiods change the autoregulatory relationship between the clock genes in mouse adipose tissue and that other regulators (e.g., feeding cues)^81^ may fine‐tune their expression to adjust to the new timing of light and/or dark. In inbred lab mice, metabolically active tissues such as the liver entrain their clock genes to feeding behavior/nutrient levels^6^, ^62^, ^82^, ^83^, ^84^, ^85^ whereas clock genes in other tissues such as lung tissue and the SCN are not entrained by feeding behavior.^64^, ^84^ Peripheral clock genes (including WAT) have been suggested to best synchronize with a “synchronous time” that is one‐quarter through the dark period,^6^ reflecting combined influences of light and feeding. In lab mice, when feeding is restricted to daylight (a 12‐h shift), hepatic clock gene expression shifts by 7–9 h,^85^ a partial phase inversion that suggests that neither photoperiod nor feeding can act alone as the zeitgeber.^85^ If adipose clocks are entrained solely to the timing of food consumption, we would expect that the clock gene expression would be similar between groups, since 24 h feeding behavior was similar. However, the relationship between these genes appears to have shifted, as their expression does not seem to be completely antiphase to one another. This supports the theory that there is a combination of cues that entrain vWAT clock genes, reflecting an altered alignment between feeding patterns and photoperiod cues.^6^

In our study, clock genes appeared to be altered by photoperiod and this was associated with effects on vWAT mass and *AdrB3* and *Ucp1* expression. Clock genes have been associated with adipose tissue metabolism through the study of various knockout mouse models^13^, ^86^, ^87^, ^88^, ^89^, ^90^ and may act as a link between altered photoperiod and vWAT physiology.

### 4.1 Study limitations

To maximize photosensitivity in our model, mice were bred from wild‐captured mice and display more variability than inbred and outbred lab‐colonized models, making the identification of weaker trends more difficult. To minimize variability, we removed outliers in an unbiased fashion by only removing data >2 standard deviations of the mean. Considering the large standard deviation of our model, very few values were removed, but this manipulation may have impacted the representation of a natural population of white‐footed mice with a more varied response to photoperiod. In addition, our study used only male mice. Understanding how female mice respond to photoperiod is also important, especially as female shift workers are more prone to increased adiposity.^5^ We intentionally only studied the impact of altered photoperiod for 4 weeks to avoid introducing bias caused by reduced caloric intake, but the short duration of the study might have missed stronger metabolic changes that happen after longer alterations in photoperiod.^26^ Other studies have shown that after 4 weeks of altered photoperiod, food consumption is affected in conjunction with changes in fat deposition, which by design we did not capture.^26^ Mice were housed at 27°C, which is in the thermoneutral zone of *Peromyscus* during the light phase.^50^ We did not adjust the housing temperature during the dark phase to account for potential diurnal changes in the thermoneutral zone, which would be expected to be higher in the dark than in the light phase.^51^ Thus, it is possible that in the dark phase there was higher energy expenditure because mice were below their thermoneutral point.^51^ By extension, since SD mice had a longer dark phase than LD mice, this subtle difference in energy expenditure may explain the difference in vWAT mass.

Translating these metabolic changes to humans must be done with caution because mice are nocturnal and may have different responses to photoperiod than humans. However, uncovering the mechanism of photoperiod‐induced regulation of adiposity will help us to understand how light at night may contribute to obesity in humans. Specifically, we show that longer photoperiod per se increases visceral WAT. For our conclusions to be translatable to humans, more clinical research should be done to study the impacts of photoperiod (especially from artificial sources) on human metabolism, feeding habits, and circadian physiology.

This novel study on the role of photoperiod at thermoneutrality in a photosensitive wild mouse model has shown that visceral and subcutaneous fat depots respond differently to environmental cues. To our knowledge, this study is the first to show that, with no physiological drive for thermal regulation, photoperiod alone can regulate adiposity. Photoperiod selectively affected vWAT mass and lipid droplet size, which suggests that scWAT might not be as photoperiod‐sensitive without the accompanying decrease in temperature. This subtle effect of photoperiod is akin to the slow fat accumulation that we see in humans over time. Visceral obesity is associated with an increased risk of many diseases (diabetes, metabolic syndrome, cardiovascular disease, cancer) more than subcutaneous adiposity, and therefore preventing visceral obesity is more important to reduce these risks.^91^ Epidemiologists have correlated an increase in obesity to increased human exposure to artificial lighting.^92^ This artificial extension of the natural solar photoperiod is becoming more prevalent as people have access to more technology.^92^ Understanding the impacts of extended and/or erratic photoperiods could help the human population better manage their circadian rhythms and reduce the incidence of obesity and related diseases.

## AUTHOR CONTRIBUTIONS

M.E. Newport, P. Wilson, and H.E. Bates contributed to study design; M.E. Newport, S. Lowes, M. Berhands, and A. Coons performed research; M.E. Newport performed data analysis; J. Bowman contributed to wild‐Peromyscus trapping; M.E. Newport and H.E. Bates wrote the manuscript; M.E. Newport, H.E. Bates, and P. Wilson edited the manuscript; and H.E. Bates and P. Wilson were principal investigators.

## CONFLICT OF INTEREST STATEMENT

The authors have no conflicts of interest to declare.

## Supporting information


Figure S1.

Figure S2.

Figure S3.

Table S1.

## Data Availability

The data that support the findings of this study are available in the Materials and Methods, Results, and/or Supplemental Material of this article.
