# Supplementary material for: Photoperiod influences visceral adiposity and the adipose molecular clock independent of temperature in wild‐derived Peromyscus leucopus
Source: FASEB Bioadv. 2025 Apr 17;7(5):e70006. doi: 10.1096/fba.2024-00115 (PMC12050962; doi:10.1096/fba.2024-00115)
Supplement: Supplementary file 1 — Figure S1. [file FBA2-7-e70006-s002.pdf]

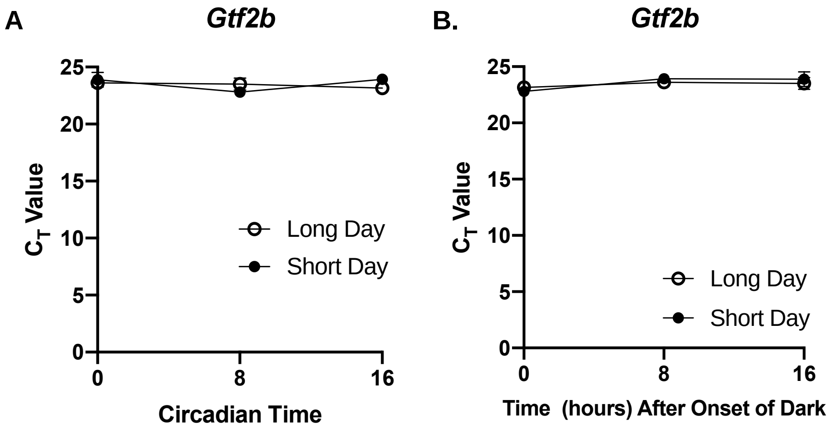

Figure S1. Gtf2b Gene Expression in Epididymal WAT. There is no effect of photoperiod or time on expression (C_T value) of Gtf2b gene when aligned to **(A) circadian time** or **(B) onset of darkness.** Data is mean ± SEM, n=10-12/group.
