# Supplementary material for: Photoperiod influences visceral adiposity and the adipose molecular clock independent of temperature in wild‐derived Peromyscus leucopus
Source: FASEB Bioadv. 2025 Apr 17;7(5):e70006. doi: 10.1096/fba.2024-00115 (PMC12050962; doi:10.1096/fba.2024-00115)
Supplement: Supplementary file 2 — Figure S2. [file FBA2-7-e70006-s004.pdf]

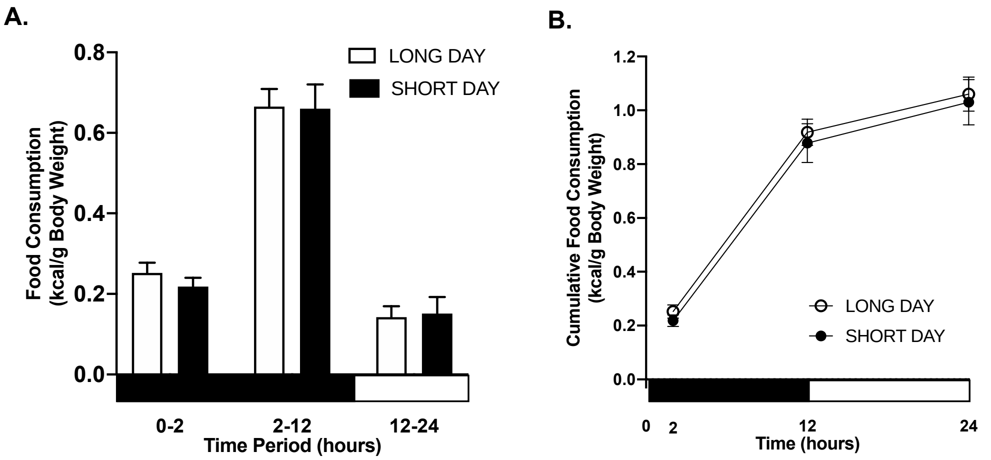

Figure S2. Baseline diurnal fasting re-feeding food intake test. Food intake was measured one week before experiment started in 12:12 lighting conditions to ensure that animals in both groups began with similar eating patterns. Feeding behaviour (A) and 24 h food consumption (B) were not impacted by photoperiod. Data is mean ± SEM, n=34/group.
