# Supplementary material for: Photoperiod influences visceral adiposity and the adipose molecular clock independent of temperature in wild‐derived Peromyscus leucopus
Source: FASEB Bioadv. 2025 Apr 17;7(5):e70006. doi: 10.1096/fba.2024-00115 (PMC12050962; doi:10.1096/fba.2024-00115)
Supplement: Supplementary file 3 — Figure S3. [file FBA2-7-e70006-s001.pdf]

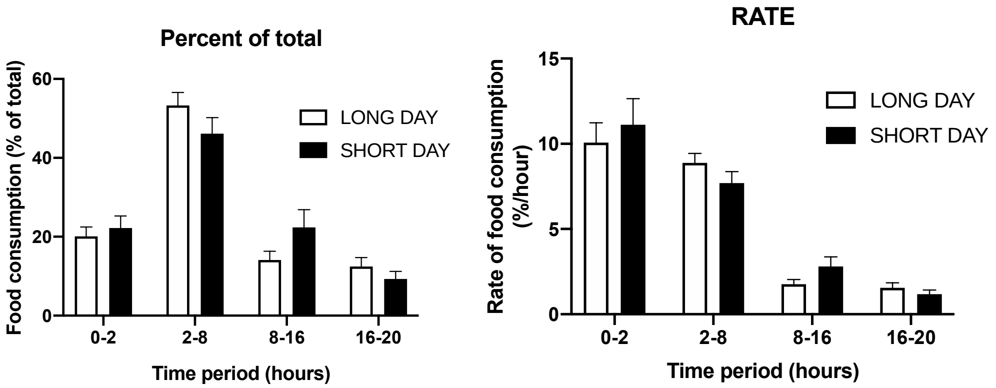

Figure S3. Experimental food intake normalized to individual 24-hour consumption. (A) Intervals measured. There were no differences in percentage of food consumed in each interval measured between photoperiod groups. **(B) Hourly rate of consumption in intervals.** Mice had the highest rate of consumption in the first two hours after food was returned in both groups. There were no differences in rate of consumption between photoperiod groups during any interval measured. Data is mean ± SEM, n= 34/group.
