# Supplementary material for: Photoperiod influences visceral adiposity and the adipose molecular clock independent of temperature in wild‐derived Peromyscus leucopus
Source: FASEB Bioadv. 2025 Apr 17;7(5):e70006. doi: 10.1096/fba.2024-00115 (PMC12050962; doi:10.1096/fba.2024-00115)
Supplement: Supplementary file 4 — Table S1. [file FBA2-7-e70006-s003.pdf]

Table S1. CytB primer sequences for genotyping

MICRO	SEQUENCE	AUTHOR
CTRL-L	5'- CAC YWT YAA CWC CCA AAG CT- 3'	38
TDKD	5'- CCT GAA GTA GGA ACC AGA TG-3'	39
